# Laxatives in Childbed

**Published:** 1889-02

**Authors:** E. Van Goidtsnoven

**Affiliations:** Atlanta, Ga.


### LAXATIVES IN CHILDBED.

E. VAN GOIDTSNOVEN, M. D., OF ATLANTA, GA.

A gravid uterus will produce :

1. A mechanical obstruction to the fœcal discharges, hence constipation.

2. An impediment to the peristaltic motion of the intestines, hence atony of their muscular wall.

3. Pressure upon the hæmorrhoidal circulation, hence piles.

Many other pathological results could be cited as concomitants of this "*interesting condition;*" but as your inquiry in the last number of the SOUTHERN MEDICAL RECORD is simply confined to laxatives in childbed, I will only mention the above as being mostly germain to the subject in question. What is wanted here is a well tried aperient rather than a purgative, a local agent *in situ*, an *ex-officio justice of the peace*, who will not only unload the bowels, but achieve this end with the least subsequent discomfort to the offspring. In the majority of cases the simple unloading of the bowels removes as by magic the above named disorders.

Look out for the milk.

Fothergill of blessed memory, lauds jalap and affirms that the milk of nursing woman is not rendered purgative by its use as it is by that of other drugs, so much in vogue nowadays. I have acted up to his recommendation for several years and with the most satisfactory results. I give 20 grains of jalap in two or more capsules, the third day after confinement, when the bowels are not spontaneously open. According to Merrell the action of jalap seems to be entirely local and to more especially effect the alimentary canal below the stomach, producing watery secretion from the intestinal surface, and stimulation of the intestinal glands.

As you see, I lay no claim to the authorship of this plan. "*Je prends le bien ou je le trouve*" and lay it humbly but not timidly before your readers.

